# Paid Employment and Mental Health in 65–74‐Year‐Olds: Analysis of National Data From 2000, 2007 and 2014

**DOI:** 10.1002/gps.6143

**Published:** 2024-09-26

**Authors:** Gayan Perera, Karen Glaser, Giorgio Di Gessa, Robert Stewart

**Affiliations:** ^1^ Department of Psychological Medicine Institute of Psychiatry, Psychology and Neuroscience King's College London London UK; ^2^ Institute of Gerontology Department of Social Science, Health & Medicine King's College London London UK; ^3^ Department of Epidemiology & Public Health Institute of Epidemiology & Health University College London London UK; ^4^ Centre for Translational Informatics South London and Maudsley NHS Foundation Trust London UK

**Keywords:** British National Surveys of Psychiatric Morbidity, common mental disorders, mental health, older people, paid employment

## Abstract

**Introduction:**

Employment rates for people aged 65 and over have been changing rapidly in many countries, but little is known about associations of employment status with mental health status and their stability over time. We therefore investigated mental health associations with employment status in 65–74‐year‐olds in three national samples.

**Methods:**

The data for these analyses were drawn from three national surveys of psychiatric morbidity among adults in England living in private households carried out in 2000, 2007, and 2014. Employment status was the primary exposure of interest. Common mental disorder (CMD) and constituent symptoms were ascertained identically in the three surveys from the revised Clinical Interview Schedule. Covariates included identical demographic, social and physical health measures.

**Results:**

A significant association between non‐employment and CMD was present in 2007 (odds ratio 2.66 [95% CI: 1.02–7.83]) but there was no significant association between non‐employment and CMD in 2000 or 2014. The largest attenuation in the association between non‐employment and CMD was seen when adjusted for physical health related factors. In combined samples, non‐employment was most strongly associated with self‐reported cognitive difficulties (OR 1.25, 1.01–1.61), depressive ideas (1.30, 1.01–1.67), worry (1.30, 1.01–1.68), and anxiety (1.27, 1.00–1.64) as constituent CMD symptoms.

**Conclusion:**

Evidence is still unclear whether employment after statutory retirement ages is associated with better mental health, and associations may be symptom‐specific. In the light of policies to encourage older workers to remain active in the labour market, more research is needed into the interrelationships between paid work and mental health, as well as other outcomes.

**Summary**

- Evidence of beneficial or neutral effects from extended working over 64 years of age on overall health status and physical health for many employees, and mixed effects on mental health.
- The stability over time of the association between paid employment and mental health is unknown but important, not only because of rapidly changing employment rates in the age range of interest over time, but also because of changes in expectations around “post‐retirement” employment, and fluidity in statutory retirement ages.
- Our study found that a significant association between non‐employment and CMD was present in 2007, but there was no significant association between non‐employment and CMD in 2000 or 2014. The largest attenuation in the association between non‐employment and CMD was seen when adjusted for physical health related factors. In combined samples, non‐employment was most strongly associated with self‐reported cognitive difficulties, depressive ideas, worry, and anxiety as constituent CMD symptoms.
- Evidence is still unclear whether employment after statutory retirement ages is associated with better mental health, and associations may be symptom‐specific.

## 1 Introduction

In common with many countries, the UK has experienced significant increases in the proportions of older workers (defined as those aged 65 and over) in the labour market, following marked declines from the late 1970s to the mid‐1990s. Specifically, the employment rate for people aged 65 and over doubled over 30 years, from 4.7% in 1992 to 11.9% in 2022, and among the 65–69 age group, employment rates started growing around the early 2000s and have continued to do so, reducing the employment gap between people aged 65–69 and younger age groups [1]. Additionally, although employment rates for men and women aged 70–74 started at a low level, they have almost doubled over the last 10 years: the employment rate for men increased from 6.9% to 12.4%, and that for women increased from 4.3% to 7.6% [1].

Many reasons may contribute to why older people decide to stay in or leave employment in later life. One study found that work motivation, health and financial situations all influenced working beyond retirement [2]. Examples of work‐related factors associated with working beyond statutory employment age are the extent to which people enjoy their work (i.e., job flexibility in working hours or less demanding jobs) and find it fulfilling [3]. Moreover, it was shown that social factors, such as having a working spouse and children to support, were positively associated with the desire to engage in bridge employment [4]. One study identified three themes as important benefits for working beyond the age of 65 [5]: increasing financial security, maintaining health, and continuing personal development. Furthermore, several theoretical perspectives provide the opportunity to gain a better understanding of the decision to prolong work participation. For example, Continuity Theory suggests that older individuals are more likely to maintain similar routines, structures and familiar social networks to those of their earlier years [6]. Therefore, the decision to prolong work participation is multifactorial, with good health an important precondition [7].

Older workers may leave the workforce due to disability, unemployment, or early retirement [8]. Several studies have found that general health problems contribute to an early exit from work [9, 10, 11]. For many people, retirement is associated with a positive change in well‐being [12, 13, 14]. However, for some, retirement arises from external pressures including redundancy, ill health, or needing to care for a sick or disabled family member [15] and, under these circumstances, may be associated with poor mental health outcomes due to the loss of a work role and social networks [16], as well as losing concordance with social norms for workforce participation and retirement age [17], demands of caring [18], or declining physical health [19].

A number of studies have examined associations between non‐employment and mental health, with many showing that earlier retirement is associated with poorer mental health. However, continued employment beyond the traditionally expected retirement age, has not always been associated with health benefits [20]. Longitudinal analyses of Health and Retirement Study data indicated that retirement led to a 6%–9% decline in mental health relative to the sample mean: greater if retirement was involuntary or at an earlier. Non‐employment due to ill health has been found to be associated with poorer mental health and physical functioning at follow‐up, whereas voluntary retirement was associated with a short period of better health [21]. A systematic review indicated evidence of beneficial or neutral effects from extended working over 64 years of age on overall health status and physical health for many employees, and mixed effects on mental health [22] while another study showed that working after retirement increased depression among older adults and aggravated their mental health [23, 24]. Marked reductions have been found in the prevalence of depressive episodes and mental fatigue following retirement [20, 25, 26], although there has been relatively little evaluation of specific depressive symptoms. Furthermore, this reduction appears to coincide with the statutory retirement age and not the timing of individual exit from work [27]. Where studies have been analysed by gender, they have found that the association between retirement and mental health may be stronger for men, particularly if they leave due to ill health [28, 29, 30]. However, women who leave the workforce to care for others may not classify themselves as retired [31]. Leaving work to undertake caring has also been associated with poor mental health outcomes [32].

The stability over time of the association between paid employment and mental health is unknown but important, not only because of rapidly changing employment rates in the age range of interest over time, but also because of changes in expectations around “post‐retirement” employment, and fluidity in statutory retirement ages, as well as secular changes in population health and the other factors that are potential determinants and/or outcomes of retirement. The relationship between employment and mental health has been investigated over many years and may well be influenced by wider regional or national contexts, such as the economic outlook and expectations of employment. Temporal stability is also an important consideration if survey findings are to be used as a basis for future policy, but has received little or no investigation. Furthermore, it is essential to understand subcomponents of mental disorders associated with older age group, so that interventions can be implemented to support such health problems. The aim of our study was to investigate the association between employment status and common mental disorder (CMD) among 65–74‐year‐olds residing in England and its stability over time, taking advantage of three national mental health surveys carried out in 2000, 2007 and 2014, and adjusting for sociodemographic and health related covariates.

## 2 Materials and Methods

### 2.1 Data and Sample

The data for these analyses were drawn from a series of national mental health surveys of adults living in private households: the British National Surveys of Psychiatric Morbidity of 2000, 2007 and 2014. The most recent surveys 2007 and 2014 survey was conducted by the National Centre for Social Research (NatCen) in collaboration with the University of Leicester while earlier surveys were carried out by the Office for National Statistics. The 2000 survey was carried out in England, Wales, and Scotland, whereas the 2007 and 2014 surveys were based in England only [33]. The lower age limit for participation was 16 years for all surveys, but the upper age limit varied: 74 for the 2000 survey, and no upper limit for the 2007 and 2014 surveys The surveys sought to use identical measures so as to maximise the comparability of results, and trends across some of these national surveys have been previously reported [34]. Data from the earliest 1993 survey were not used because a 64‐year upper age cut‐off had been imposed and a decision was made to focus on the 60–74 age range for the other three surveys as employed status in older (75+) age ranges was insufficiently prevalent and likely to vary in its determinants.

The sampling methodology was comparable across the three surveys, which employed independent random sampling across the geographic areas in question, not seeking previous participants or sampling from identical areas. In each, primary sampling units (postal sectors) were selected from the Small Users Postcode Address File, stratified for region and social‐class composition to generate a nationally representative sample. Households were randomly selected from within each sampling unit, and in households containing at least one member in the age range for that survey, one person was randomly selected and invited to participate. Each person was only interviewed once. In 2000, 8580 participants were interviewed (67.1% participation); in 2007, 7403 participants were interviewed (56.2%); and in 2014, 7508 participants were interviewed (57.2%). Our analysis focused on participants aged 65–74 years, and restricted the 2000 survey to residents in England, in order to maximise comparability across the three surveys. The analysed sample contained 3029 participants from the three surveys combined (1056 in 2000, 1003 in 2007 and 970 in 2014).

### 2.2 Variables

Employment status was the primary exposure of interest. In each survey participants were asked whether they had carried out any paid work in the preceding week, with responses to further questions on income earned from employment or self‐employment used to cross‐check employment status categorisation. Reasons for not being in paid work were enquired about in all age groups but there was insufficient variability in responses in this sample (> 97% stating retirement as a reason) to warrant analysis.

Common mental disorder (CMD), as the primary outcome, was ascertained identically in the three surveys from the revised Clinical Interview Schedule (CIS‐R) [35], which is a widely used, fully‐structured questionnaire with stem and supplementary questions enquiring in detail about the following 14 symptoms in the past week: somatic, fatigue, concentration/forgetfulness, sleep problems, irritability, health worry, depression, depressive ideas, general worry, anxiety, phobias, panic, compulsions and obsessions. Each symptom schedule generates a 0–4 or 0–5 score based on frequency, duration, and severity in the preceding week. A summed CIS‐R score of 12 of more is conventionally taken to indicate CMD on the basis of the presence of significant neurotic symptoms of a level likely to impact on day‐to‐day functioning, cause distress, and be responsive to treatment [35].

Based on previous research findings, we extracted covariates which were recognised to be associated with either or both paid work and CMD, and which had been ascertained in an identical manner in all surveys. These included the following socio‐demographic covariates: age, gender, marital status, education, social class, housing tenure, financial difficulty, smoking status, and physical health. Participation in paid work tends not to decrease constantly with age so we used a categorical indicator of age group (65–69; 70–74). Marital status was grouped into four categories: divorced/separated, married/cohabiting, single, and widowed. Respondents' highest educational qualifications were grouped into three categories: (i) A‐level and above (implying a school leaving age of at least 18), (ii) GCSE/GCE/O‐level (implying a school leaving age of 16), and (iii) no qualifications. Social class was determined by the respondents' primary occupation or most recent occupation (categorised into the I, II, IIIn, IIIM, IV, and V Registrar General classification; those categorised as “Armed forces” (9 respondents) or “Never worked” (89 respondents) were excluded). Housing tenure was categorised into four groups: (i) owned with a mortgage, (ii) owned outright, (iii) privately rented, and (iv) social housing. In addition, serious financial difficulty was defined as either a reported financial crisis in the previous 6 months or being behind with any payments. Self‐reported smoking status was grouped into never smoked, ex‐smoker and current smoker categories. Self‐reported physical conditions had been ascertained using different approaches in the surveys and these measures were considered too heterogeneous to use in this analysis. However, in all surveys participants were asked whether they had consulted a general practitioner in the previous 12 months for a physical health problem which was coded as a binary variable, and this was supplemented by scores for an activities of daily living (ADL) scale, identically administered in all surveys and enquiring about difficulties in the following domains on a 0–2 scale: (i) personal care; (ii) using transport; (iii) medical care; (iv) household activities; (v) practical activities; (vi) paper work; (vii) money. Because of the distribution of scores in the sample age group, a binary variable was created indicating some difficulty or lot of difficulty for at least one ADL domain.

### 2.3 Statistical Analysis

Consistent with previous cross‐survey publications, analyses were not weighted, as surveys used different weighting, precluding comparisons of estimates over time. We first assessed bivariate associations between all covariates and paid employment, followed by unadjusted logistic regression analyses, assessing the bivariate associations between our key independent variable and all covariates and CMD. Multivariable logistic regression models were carried out to investigate the association between employment status and CMD at various levels of adjustments including demographics, socioeconomic status, and physical health measures. Multivariate regression analyses were carried out and assessed using pseudo *R* ^2^. Likelihood ratio tests were conducted to investigate associations between survey year and employment status. Finally, regression analyses were repeated for each individual CMD subscale, defined as binary variables on the basis of a 2+ score, as is standard for this instrument.

## 3 Results

Characteristics of the employed and non‐employed groups are summarised in Table 1. The prevalences of paid employment for the sample age group in the 2000, 2007 and 2014 surveys were 10.0% and 13.2% and 15.1% respectively. Of those in employment, the proportions reporting working full time increased from 14.2% in 2000 to 27.3% in 2007 and to 37.7% in 2014. The overall prevalence of CMD was 8.7% in 2000, 10.4% in 2007 and 9.9% in 2014, and was lower in the employed group in all surveys. The groups in paid employment had a consistently higher proportion of males than those in non‐employment in all surveys, and nearly two thirds of those in paid employment were married or cohabiting, higher than in the non‐employed group in all surveys.

**TABLE 1:** Description of the sample by employment status and year of survey in 65–74‐year‐olds.

Variable	2000		2007		2014	
	In paid employment	Not in paid employment	In paid employment	Not in paid employment	In paid employment	Not in paid employment
Number	106	950	132	871	146	824
Age (years)						
65–69	75 (70.8)	483 (50.8)	96 (72.7)	421 (48.3)	101 (69.2)	441 (53.5)
70–74	31 (29.2)	467 (49.2)	36 (27.3)	450 (51.7)	45 (30.8)	383 (46.5)
Gender						
Male	58 (54.7)	396 (41.7)	78 (59.1)	379 (43.5)	83 (56.8)	349 (42.4)
Female	48 (45.3)	554 (58.3)	54 (40.9)	492 (56.5)	63 (43.2)	475 (57.6)
Marital status						
Divorced/separated	14 (13.2)	80 (8.4)	27 (20.5)	113 (13.0)	30 (20.5)	124 (15)
Married/cohabiting	65 (61.3)	545 (57.4)	85 (64.4)	458 (52.6)	100 (68.5)	625 (75.8)
Single	5 (4.7)	64 (6.7)	6 (4.5)	59 (6.8)	12 (8.2)	57 (6.9)
Widowed	22 (20.8)	261 (27.5)	14 (10.6)	241 (27.7)	4 (2.7)	18 (2.2)
Highest qualification						
A‐level or above	51 (48.1)	482 (50.7)	47 (35.6)	185 (21.2)	46 (31.5)	225 (27.3)
GCSE/GCE/O‐level/other	31 (29.2)	286 (30.1)	39 (29.5)	159 (18.3)	34 (23.3)	166 (20.1)
No qualifications	24 (22.6)	178 (18.7)	41 (31.1)	501 (57.5)	66 (45.2)	432 (52.4)
Social class by occupation						
Professional/managerial/clerical	51 (48.1)	522 (54.9)	89 (67.4)	465 (53.4)	65 (44.5)	285 (34.6)
Skilled/partly skilled	32 (30.2)	346 (36.4)	38 (28.8)	324 (37.2)	69 (47.3)	479 (58.1)
Unskilled	11 (10.4)	77 (8.1)	4 (3.0)	72 (8.3)	12 (8.2)	48 (5.8)
Housing tenure						
Owned with a mortgage	16 (15.1)	59 (6.2)	24 (18.2)	39 (4.5)	20 (13.7)	39 (4.7)
Owned outright	64 (60.4)	655 (68.9)	87 (65.9)	606 (69.6)	103 (70.5)	613 (74.4)
Private or other renter	10 (9.4)	206 (21.7)	10 (7.6)	42 (4.8)	17 (11.6)	153 (18.6)
Social housing	5 (4.7)	29 (3.1)	11 (8.3)	179 (20.6)	6 (4.1)	19 (2.3)
Difficulties in ADL present	25 (23.6)	404 (42.5)	41 (31.1)	475 (54.5)	21 (14.4)	289 (35.1)
Physical health consultation	66 (62.3)	700 (73.7)	94 (71.2)	672 (77.2)	88 (60.3)	572 (69.4)
Smoking status						
No smoker	23 (21.7)	222 (23.4)	34 (25.8)	279 (32.0)	54 (37.0)	261 (31.7)
Ex‐smoker	66 (62.3)	578 (60.8)	78 (59.1)	453 (52.0)	79 (54.1)	463 (56.2)
Current smoker	15 (14.2)	150 (15.8)	20 (15.2)	139 (16.0)	13 (8.9)	98 (11.9)
Common mental disorder present	6 (5.7)	86 (9.1)	4 (3.0)	100 (11.5)	11 (7.5)	85 (10.3)
CMD subcomponents present						
Somatic	1 (0.9)	43 (4.5)	9 (6.8)	36 (4.1)	5 (3.4)	33 (4)
Fatigue	17 (16)	215 (22.6)	20 (15.2)	240 (27.6)	25 (17.1)	183 (22.2)
Forgetful	2 (1.9)	60 (6.3)	2 (1.5)	67 (7.7)	5 (3.4)	42 (5.1)
Sleep	23 (21.7)	291 (30.6)	26 (19.7)	301 (34.6)	41 (28.1)	247 (30.0)
Irritability	6 (5.7)	67 (7.1)	4 (3.0)	58 (6.7)	8 (5.5)	57 (6.9)
Worry about physical health	7 (6.6)	65 (6.8)	3 (2.3)	71 (8.2)	14 (9.6)	66 (8)
Depression^a^	3 (2.8)	70 (7.4)	7 (5.3)	84 (9.6)	10 (6.8)	65 (7.9)
Depressive ideas^a^	3 (2.8)	35 (3.7)	3 (2.3)	53 (6.1)	3 (2.1)	42 (5.1)
Worry	9 (8.5)	90 (9.5)	12 (9.1)	98 (11.3)	12 (8.2)	96 (11.7)
Anxiety	9 (8.5)	44 (4.6)	1 (0.8)	57 (6.5)	8 (5.5)	59 (7.2)
Phobias	3 (2.8)	22 (2.3)	2 (1.5)	19 (2.2)	5 (3.4)	20 (2.4)
Panic	1 (0.9)	6 (0.6)	0 (0.0)	13 (1.5)	0 (0)	5 (0.6)
Compulsions	1 (0.9)	20 (2.1)	2 (1.5)	20 (2.3)	1 (0.7)	16 (1.9)
Obsessions	5 (4.7)	36 (3.8)	4 (3.0)	30 (3.4)	6 (4.1)	38 (4.6)
Type of employment						
Full time	15 (14.2)		36 (27.3)		55 (37.7)	
Part time	79 (74.5)		96 (72.7)		91 (62.3)	
Non‐employed		950 (100.0)		871 (100.0)		824 (100.0)

^a^ Depression subscale items focus on low mood and anhedonia; depressive ideas items encompass diurnal variation, loss of libido, psychomotor disturbance, guilt, worthlessness and helplessness.

Unadjusted associations between covariates and CMD are summarised in Table 2. In all three surveys, CMD was associated with being female, presence of ADL impairment and physical health condition. Non‐employment was associated with CMD in the 2007 survey but not in the other 2 years, but no evidence was found for a statistical interaction between employment status and survey year (likelihood ratio test: chi squared 0.22, *p*‐value 0.640).

**TABLE 2:** Unadjusted analyses of factors associated with common mental disorder (CMD) in 65–74‐year‐olds in the three national surveys.

Exposure	OR (95% CI)	*p* value	OR (95% CI)	*p* value	OR (95% CI)	*p* value
	2000 survey		2007 survey		2014 survey	
Age (years)						
65–69	Ref.		Ref.		Ref.	
70–74	0.90 (0.58, 1.32)	0.58	0.96 (0.65, 1.43)	0.85	0.60 (0.39, 0.94)	0.03
Female gender	1.93 (1.28, 2.93)	< 0.001	2.02 (1.31, 3.10)	< 0.001	1.69 (1.08, 2.63)	0.02
Marital status						
Married/cohabiting	Ref.		Ref.		Ref.	
Single	2.70 (1.58, 4.61)	< 0.001	2.70 (1.53, 4.76)	< 0.001	1.64 (0.80, 3.35)	0.18
Widowed	0.40 (0.12, 1.31)	0.13	3.22 (1.58, 6.56)	< 0.001	2.15 (0.71, 6.53)	0.18
Divorced/separated	1.25 (0.80, 1.94)	0.33	2.51 (1.56, 4.06)	< 0.001	0.97 (0.53, 1.77)	0.91
Highest level of qualification						
A/Level and above	Ref.		Ref.		Ref.	
GCSE/GCE/O‐level/other	1.85 (0.92, 3.73)	0.09	1.24 (0.61, 2.49)	0.55	1.11 (0.57, 2.16)	0.77
No qualifications	1.01 (0.97, 1.06)	0.99	2.46 (0.95, 6.36)	0.06	1.54 (0.91, 2.60)	0.11
Social class by occupation						
Professional/managerial/clerical	Ref.		Ref.		Ref.	
Skilled/partly‐skilled occupations	1.47 (0.97, 2.23)	0.07	1.09 (0.70, 1.68)	0.71	1.24 (0.79, 1.96)	0.35
Unskilled	2.88 (1.64, 5.08)	< 0.001	1.54 (0.77, 3.10)	0.22	0.94 (0.35, 2.51)	0.90
Housing tenure						
Owned outright	Ref.		Ref.		Ref.	
Owned with a mortgage	1.40 (0.67, 2.93)	0.37	1.70 (0.77, 3.75)	0.19	2.97 (1.41, 6.25)	< 0.001
Private or other renter	2.48 (1.64, 3.76)	< 0.001	2.57 (1.64, 4.03)	< 0.001	1.99 (0.57, 6.88)	0.28
Social housing	1.56 (0.54, 4.54)	0.42	1.35 (0.56, 3.29)	0.51	4.05 (2.52, 6.49)	< 0.001
Not in paid employment in the preceding week	1.66 (0.71, 3.89)	0.25	4.15 (1.50, 11.5)	0.01	1.41 (0.73, 2.73)	0.30
Difficulties in ADL present	6.12 (3.67, 10.2)	< 0.001	7.14 (4.01, 12.7)	< 0.001	4.60 (2.96, 7.17)	< 0.001
Physical health consultation	4.34 (2.07, 9.08)	< 0.001	2.84 (1.49, 5.40)	< 0.001	2.34 (1.36, 4.03)	< 0.001
Smoking status						
No smoker	Ref.		Ref.		Ref.	
Ex‐smoker	0.79 (0.50, 1.25)	0.32	0.66 (0.42, 1.04)	0.07	1.11 (0.68, 1.81)	0.68
Current smoker	1.24 (0.71, 2.18)	0.45	1.10 (0.63, 1.93)	0.73	2.07 (1.09, 3.92)	0.03

The association between non‐employment and CMD was further investigated with successive adjustments in multiple logistic regression models and findings are summarised in Table 3. Non‐employment remained significantly associated with CMD when adjusted for socio‐demographic, socio‐economic and health factors in 2007, but not in 2000 or 2014. Overall model strengths were similar across all three survey years with highest pseudo *R* ^2^ coefficients for the models which included health factors. Considering gender differences in the association between non‐employment and CMD, numbers were insufficient to analyse separately between surveys. Combining the surveys, fully adjusted odds ratios for CMD and non‐employment were 1.40 (95% CI: 0.60–3.26) for females, and 3.63 (1.02–16.0) for males.

**TABLE 3:** Adjusted associations of non‐employment with CMD among 65–74‐year‐olds in the three national surveys.

Adjustments	2000 (*n* = 1056)			2007 (*n* = 1003)			2014 (*n* = 970)		
	OR	*p* value	Pseudo *R* ^2^	OR	*p* value	Pseudo *R* ^2^	OR	*p* value	Pseudo *R* ^2^
Model 1: Age, gender and marital status	1.76 (0.74–4.20)	0.20	0.04	4.10 (1.43–11.3)	0.01	0.06	1.47 (0.76, 2.88)	0.26	0.03
Model 2: Model 1+ education, social class, housing tenure	1.88 (0.71–4.94)	0.20	0.07	3.94 (1.35–11.5)	0.01	0.07	1.38 (0.69, 2.79)	0.37	0.08
Model 3: Model 2+ smoking status, difficulties in ADL + seen a doctor in the past 12 months	1.22 (0.46–3.27)	0.69	0.16	2.66 (1.02–7.83)	0.04	0.15	0.99 (0.48, 2.06)	0.98	0.16

Table 4 summarises associations between subcomponents of CMD in combined surveys adjusted successively. Only cognitive disturbances, depressive ideas, and worry/anxiety were significantly associated with non‐employment exposure in fully adjusted models. Depression, somatic problems, fatigue, irritability, panic, compulsions, and obsessions as outcomes were associated with non‐employment when adjusted for sociodemographic and socioeconomic factors, but the associations were reduced substantially in strength when adjusted for physical health factors. No marked differences in findings were observed following stratification by gender (data not shown).

**TABLE 4:** Odds ratios of non‐employment association with CMD subcomponents in combined surveys among 65–74‐year‐olds, successively adjusted.

CMD subcomponent	Association with non‐employed status (OR, 95% CI, with successive adjustments) (*n* = 3266)					
	Model 1	Pseudo *R* ^2^	Model 2	Pseudo *R* ^2^	Model 3	Pseudo *R* ^2^
Somatic	1.28 (1.01, 1.61), 0.04	0.09	1.35 (1.05, 1.73), 0.02	0.20	1.25 (0.97, 1.61), 0.09	0.21
Fatigue	1.32 (1.04, 1.68), 0.02	0.07	1.38 (1.07, 1.78), 0.01	0.16	1.22 (0.94, 1.58), 0.14	0.18
Forgetful	1.30 (1.03, 1.64), 0.03	0.09	1.36 (1.05, 1.74), 0.02	0.19	1.25 (1.01, 1.61), 0.04	0.20
Sleep	1.22 (0.95, 1.57), 0.12	0.07	1.26 (0.97, 1.64), 0.08	0.13	1.12 (0.86, 1.47), 0.40	0.16
Irritability	1.30 (1.03, 1.64), 0.03	0.09	1.37 (1.07, 1.76), 0.01	0.19	1.27 (0.99, 1.64), 0.06	0.20
Worry about physical health	1.23 (0.98, 1.56), 0.08	0.09	1.28 (0.99, 1.64), 0.06	0.19	1.16 (0.89, 1.49), 0.27	0.20
Depression^a^	1.29 (1.02, 1.63), 0.03	0.09	1.34 (1.04, 1.72), 0.02	0.20	1.23 (0.95, 1.59), 0.11	0.20
Depressive ideas^a^	1.34 (1.06, 1.68), 0.01	0.09	1.41 (1.10, 1.81), 0.01	0.19	1.30 (1.01, 1.67), 0.04	0.21
Worry	1.33 (1.05, 1.68), 0.02	0.08	1.40 (1.09, 1.81), 0.01	0.19	1.30 (1.01, 1.68), 0.04	0.20
Anxiety	1.30 (1.03, 1.63), 0.03	0.08	1.38 (1.07, 1.77), 0.01	0.19	1.27 (1.00, 1.64), 0.05	0.20
Phobias	1.25 (0.99, 1.57), 0.06	0.09	1.31 (1.02, 1.69), 0.04	0.20	1.22 (0.95, 1.57), 0.13	0.21
Panic	1.28 (1.02, 1.62), 0.03	0.09	1.36 (1.06, 1.75), 0.02	0.21	1.27 (0.98, 1.64), 0.07	0.22
Compulsions	1.30 (1.03, 1.63), 0.03	0.09	1.37 (1.07, 1.77), 0.01	0.20	1.28 (0.99, 1.65), 0.06	0.21
Obsessions	1.28 (1.01, 1.61), 0.04	0.09	1.35 (1.05, 1.73), 0.02	0.20	1.25 (0.97, 1.61), 0.09	0.21

*Note:* Model 1 adjusted for Age, gender and marital status; Model 2 adjusted for adjustments in Model 1+ education, social class, housing tenure; Model 3 adjusted for adjustments in Model 2+ smoking status, difficulties in ADL + seen a doctor in the past 12 months.

^a^ Depression subscale items focus on low mood and anhedonia; depressive ideas items encompass diurnal variation, loss of libido, psychomotor disturbance, guilt, worthlessness and helplessness.

## 4 Discussion

This analysis of nationally representative survey data assessed the relationship between non‐employment and CMD in people aged 65–74 years. While we found a clear and independent association in the 2007 survey, there was no significant association in 2000 or in 2014. Considering component symptoms of CMD, non‐employment was primarily associated with self‐reported cognitive difficulties, depressive ideas, worry and anxiety in the combined survey samples and associations were relatively weak for most other CMD component symptoms; furthermore, those with depression, somatic, fatigue, irritability, panic, compulsions, and obsessions appeared substantially confounded by worse physical health.

A recent study in an Australian population found that retirement and unemployment were associated with higher psychological distress in men and women aged 45–64 but only in men aged 65–74 years [36]. Of cross‐sectional studies, one found that unemployed retirees in this age group were more likely to report poor psychological well‐being, but another found no association between retirement and measures of mental health or psychological distress among people aged 65 years and over [37]. These discrepancies have not been resolved by longitudinal investigations, which have typically focused on occupational cohorts. While some studies have reported increased depression and/or anxiety symptoms [38], others have found improved mental health following retirement [39]. Factors that may moderate the relationship between retirement and mental health include gender [40] but our study was not able to demonstrate this to a statistically significant extent. It has been suggested that mental health might improve for people who retire, because of reduced pressures and demands [39]. A recent European report found that over 30% of retirees would have preferred to keep working past the time they retired, with “push factors” such as job loss and ill health driving early retirement rather than “pull factors” such as incentive schemes [41]. However, others argue that retirement by people with poor physical health will be associated with worse mental health due to the combined effects of illness [19] and loss of role [21] and that involuntary job loss worsens mental health [42].

Overall, our findings did not consistently show an association between non‐employment and CMD in this post‐retirement age range, as this association was only statistically significant in one of three surveys analysed. Differences in sample size or measurement accuracy between surveys are unlikely to account for this heterogeneity, as these were relatively consistent. Furthermore, other correlates of CMD were also consistent in all three samples (e.g., associations with gender, housing tenure and physical health), although interestingly there were also stronger associations of widowed or divorced/separated marital status with CMD in 2007. An obvious potential reason for different findings in 2007 compared to 2000/2014 was the co‐occurrence of the financial crisis in the UK, and globally, in that year. It was not possible to investigate this further using the data available; however, it does at least raise the possibility that broader economic contexts might modify individual‐level associations of post‐retirement occupation with mental health, potentially accounting for at least some of the between‐study heterogeneity observed.

Our study also found particular associations between non‐employment and self‐reported cognitive difficulties, depressive ideas, worry and anxiety as component CMD symptoms in these 65–74‐year‐old samples, suggesting that heterogeneous findings might also reflect differences in symptom capture between measurements. Regarding cognitive functioning, Adam et al. [43] found a strong association between unemployment and lower cognitive function among 65‐ to 74‐year‐olds, Rohwedder and Willis [44] found a strong relationship between unemployment due to retirement and lower cognition, while Coe et al. [45] found no association between cognitive function and retirement. However, these all investigated objectively measured cognitive function rather than self‐reported measures. Better perceived cognitive function, less anxiety, less depressive ideas, and less worry in people who work beyond retirement age might well reflect the regularity of lifestyle and socialisation facilitated by the working day, although reverse effects (depressive ideas, worry and anxiety and perceived cognitive failures precipitating withdrawal from the workforce) should also be considered. Leaving employment in older age, for any reason, has a substantial negative impact on social engagement and self‐esteem [46] and may impact on loneliness, which in turn may impact on poor health behaviours, anxiety, fatigue, and cognitive decline [47, 48]. The combination of frailty and loneliness due to loss of occupation may increase risk of depressive symptoms [49], and these findings should be considered in the development of social programs for older adults to reduce the risk of depression, and aid older adults in maintaining their functional status.

Although cognitive disturbance, depressive ideas, worry, and anxiety are components of CMD in most schedules, the lack of independent associations between employment status and other CMD symptoms, along with the inconsistency between the survey years does not support a strong link between retirement and mental health. Although higher scores on the CIS‐R somatic, irritability, depression, panic, compulsions, obsessions, and fatigue subscales were associated with non‐employment independent of social and demographic covariates, these were reduced substantially in strength following adjustment for physical health factors. The relationship of employment status to CMD symptoms appears therefore to be better explained by lifestyle and physical health rather than an impact of retirement on mental health per se.

Strengths of the analysis presented here include the relatively large samples, and the unique data resource from relatively consistent samples and measurements over three national surveys covering 14 years, as well as a schedule permitting investigation of individual component CMD symptoms. However, important limitations should also be taken into consideration. One limitation could be the validity and utility as well as the potential instability and error associated with using the construct of CMD in older people with comorbidity, although the 65–74 years age range was focused on as a period when comorbidity is less of a confounder than in older age groups with higher levels of frailty. The CIS‐R instrument has been used widely including internationally in India [50] and in Ethiopia [51]; however, one study found that clinical databases are likely to yield underestimates of the burden of CMD in the adolescent population [52]. Furthermore, among the Whitehall II participants age 58 to 80, authors found that the CIS‐R was feasible and has good validity as a measure of any mental disorder and depression [53]. Although a number of measurements were equivalent across surveys, some were not, and this limited the variables that could be included. In addition, the varying response rates over time should be borne in mind when drawing interpretations. Since these data are cross‐sectional, it is not possible to determine whether mental health factors preceded or precipitated non‐employment. For instance, a person's retirement decision can be seen as a process, and the context surrounding such decisions are fragmentary when retirement is considered cross‐sectionally. Preceding events and experiences, such as difficult work histories, decreasing financial resources, poor access to adequate retirement planning information, disruptions to health, loss of leisure activities and poor marital quality are all important resources, which can influence a person's retirement and psychological well‐being [40, 54, 55], and cannot be explored fully in this study. Finally, we did not attempt to investigate employment status in older age ranges, as this was only available for two of the surveys and we felt would be insufficiently prevalent to analyse reliably (e.g., numbers of 70–74–year‐olds in paid employment were only 31, 36, and 45 across the respective surveys), as well as being potentially heterogeneous in its determinants and outcomes.

In conclusion, after investigating the relationship between non‐employment and CMD in people aged 65–74 years using an analysis of data from three nationally representative surveys with comparable samples and measurements, we found a clear and independent association in the 2007 survey, there was no significant association in 2000 or in 2014, indicating that associations of non‐employment and mental health in this age group are not consistent at different times and therefore potentially modified by changing and/or geographically varying societal or economic environments. Considering component symptoms of CMD, non‐employment was primarily associated with self‐reported cognitive difficulties, depressive ideas, worry and anxiety in the combined survey samples and associations were relatively weak for most other CMD component symptoms; furthermore, those with depression, somatic, fatigue, irritability, panic, compulsions, and obsessions appeared substantially confounded by worse physical health. Therefore, evidence is still unclear whether employment after statutory retirement ages is associated with better general mental health, as associations may be symptom‐specific. These findings need to be considered in policy decisions around health and retirement, as well as in individual‐level clinical practice with patients in the “late middle age” range. In the light of policies to encourage older workers to remain active in the labour market, more research is needed into these interrelationships between paid work and mental health, as well as other outcomes, and policy makers need to consider the potential for broader external influences, including the timing of implementation.

## Author Contributions

Analyses were carried out by G.P. and designed together with R.S., K.G., and G.D.G. The manuscript was finalised by R.S. and G.P. with substantial text contributions and further comments and significant input from all remaining co‐authors.

## Conflicts of Interest

All authors received support from the Economic and Social Research Council (ESRC) and the Medical Research Council (MRC) through an Extended Working Lives Consortia Grant (ES/L002825/1). It is also partly supported by the ESRC Centre for Society and Mental Health at King's College London (ES/S012567/1 to K.G.). Robert Stewart declares research support received in the last 3 years from Janssen, GSK, and Takeda.

## Data Availability

Access to the 2000 and 2007 APMS dataset is via the UK Data Service: https://beta.ukdataservice.ac.uk/datacatalogue/studies/study?id=6379. Access to the 2014 APMS dataset is by formal application to NHS Digital.

## References

[1] Department for Work and Pension , “Employment Statistics for Workers Aged 50 and Over, by 5‐Year Age Bands and Gender” (2015), https://assets.publishing.service.gov.uk/media/5a806b8c40f0b62305b8b0c5/employment‐stats‐workers‐aged‐50‐and‐over‐1984‐2015.pdf.

[2] A. de Wind , S. van der Pas , B. M. Blatter , and A. J. van der Beek , “A Life Course Perspective on Working Beyond Retirement: Results From a Longitudinal Study in the Netherlands,” BMC Public Health 16, no. 1 (2016): 1–12, 10.1186/s12889-016-3174-y. 27287303 PMC4902896

[3] T. A. Beehr , M. M. Bennett , K. S. Shultz , and G. A. Adams , “Examining Retirement From a Multi‐Level Perspective,” in Aging and Work in the 21st Century, eds. K. S. Shultz and G. A. Adams (Mahwah: Erlbaum, 2007), 277–302.

[4] S. Kim and D. C. Feldman , “Working in Retirement: The Antecedents of Bridge Employment and Its Consequences for Quality of Life in Retirement,” Academy of Management Journal 43, no. 6 (2000): 1195–1210, 10.2307/1556345.

[5] F. A. Reynolds , A. Farrow , and A. Blank , “‘Otherwise It Would Be Nothing but Cruises’: Exploring the Subjective Benefits of Working beyond 65,” International Journal of Applied Logistics 7, no. 1 (2012): 79–106, 10.3384/ijal.1652-8670.1271.

[6] R. C. Atchley , “A Continuity Theory of Normal Aging,” Gerontologist 29, no. 2 (1989): 183–190, 10.1093/geront/29.2.183. 2519525

[7] R. Sewdas , A. de Wind , L. G. L. van der Zwaan , et al., “Why Older Workers Work Beyond the Retirement Age: A Qualitative Study,” BMC Public Health 17, no. 1 (August 2017): 672, 10.1186/s12889-017-4675-z. 28830399 PMC5567892

[8] G. Di Gessa , L. M. Corna , L. G. Platts , et al., “Is Being in Paid Work Beyond State Pension Age Beneficial for Health? Evidence From England Using a Life‐Course Approach,” Journal of Epidemiology & Community Health 71, no. 5 (May 2017): 431–438, 10.1136/jech-2016-208086. 27940656 PMC5484027

[9] S. Vickerstaff , “Older Workers: The ‘Unavoidable Obligation’ of Extending Our Working Lives?,” Sociology Compass 4, no. 10 (October 2010): 869–879, 10.1111/j.1751-9020.2010.00322.x.

[10] G. Mein , P. Martikainen , S. A. Stansfeld , E. J. Brunner , R. Fuhrer , and M. G. Marmot , “Predictors of Early Retirement in British Civil Servants,” Age and Ageing 29, no. 6 (November 2000): 529–536, 10.1093/ageing/29.6.529. 11191246

[11] C. W. Monden , “Current and Lifetime Exposure to Working Conditions. Do They Explain Educational Differences in Subjective Health?,” Social Science & Medicine 60, no. 11 (June 2005): 2465–2476, 10.1016/j.socscimed.2004.11.017. 15814172

[12] J. E. Byles , M. Tavener , I. Robinson , et al., “Transforming Retirement: New Definitions of Life After Work,” Journal of Women & Aging 25, no. 1 (2013): 24–44, 10.1080/08952841.2012.717855. 23199311

[13] M. A. Hardy , “The Transformation of Retirement in Twentieth‐Century America: From Discontent to Satisfaction,” Generations 26, no. 2 (2002): 9–16.

[14] J. E. Kim and P. Moen , “Is Retirement Good or Bad for Subjective Well‐Being?,” Current Directions in Psychological Science 10, no. 3 (2001): 83–86, 10.1111/1467-8721.00121.

[15] S. M. Alavinia and A. Burdorf , “Unemployment and Retirement and Ill‐Health: A Cross‐Sectional Analysis Across European Countries,” International Archives of Occupational and Environmental Health 82, no. 1 (2008): 39–45, 10.1007/s00420-008-0304-6. 18264715 PMC2467501

[16] H. Van Solinge and K. Henkens , “Adjustment to and Satisfaction With Retirement: Two of a Kind?,” Psychology and Aging 23, no. 2 (2008): 422–434, 10.1037/0882-7974.23.2.422. 18573015

[17] P. Clarke , V. Marshall , J. House , and P. Lantz , “The Social Structuring of Mental Health Over the Adult Life Course: Advancing Theory in the Sociology of Aging,” Social Forces 89, no. 4 (2011): 1287–1313, 10.1093/sf/89.4.1287. PMC3210581 22081728

[18] B. Dow and C. Meyer , “Caring and Retirement: Crossroads and Consequences,” International Journal of Health Services 40, no. 4 (2010): 645–665, 10.2190/HS.40.4.e. 21058536

[19] J. E. Byles , I. Robinson , E. Banks , et al., “Psychological Distress and Comorbid Physical Conditions: Disease or Disability?,” Depression and Anxiety 31, no. 6 (2013): 524–532, 10.1002/da.22162. 23922120

[20] E. Calvo , N. Sarkisian , and C. R. Tamborini , “Causal Effects of Retirement Timing on Subjective Physical and Emotional Health,” Journals of Gerontology Series B: Psychological Sciences and Social Sciences 68, no. 1 (January 2013): 73–84, 10.1093/geronb/gbs097. 23149431

[21] M. Jokela , J. E. Ferrie , D. Gimeno , et al., “From Midlife to Early Old Age: Health Trajectories Associated With Retirement,” Epidemiology 21, no. 3 (May 2010): 284–290, 10.1097/EDE.0b013e3181d61f53. 20220519 PMC3204317

[22] S. Baxter , L. Blank , A. Cantrell , and E. Goyder , “Is Working in Later Life Good for Your Health? A Systematic Review of Health Outcomes Resulting From Extended Working Lives,” BMC Public Health 21 (December 2021): 1, 10.1186/s12889-021-11423-2. 34238265 PMC8268509

[23] L. Xie , Y. D. Yao , L. L. Tang , et al., “Effect of Working After Retirement on the Mental Health of Older People: Evidence From China,” Frontiers in Psychiatry 12 (September 2021): 73137, 10.3389/fpsyt.2021.731378. PMC8505967 34650455

[24] L. Carrino , K. Glaser , and M. Avendano , “Later Retirement, Job Strain, and Health: Evidence From the New State Pension Age in the United Kingdom,” Health Economics 29, no. 8 (August 2020): 891–912, 10.1002/hec.4025. 32396995

[25] H. Westerlund , A. Nyberg , P. Bernin , et al., “Managerial Leadership Is Associated With Employee Stress, Health, and Sickness Absence Independently of the Demand‐Control‐Support Model,” Work 37, no. 1 (2010): 71–79, 10.3233/WOR-2010-1058. 20858989

[26] A. Odone , V. Gianfredi , G. P. Vigezzi , et al., “Does Retirement Trigger Depressive Symptoms? A Systematic Review and Meta‐Analysis,” Epidemiology and Psychiatric Sciences 30 (December 2021): e77, 10.1017/S2045796021000627. 35048820 PMC8679838

[27] E. Villamil , F. A. Huppert , and D. Melzer , “Low Prevalence of Depression and Anxiety Is Linked to Statutory Retirement Ages Rather Than Personal Work Exit: A National Survey,” Psychological Medicine 36, no. 7 (2006): 999–1009, 10.1017/S0033291706007719. 16650345

[28] L. G. Platts , L. M. Corna , D. Worts , P. McDonough , D. Price , and K. Glaser , “Returns to Work After Retirement: A Prospective Study of Unretirement in the United Kingdom,” Ageing and Society 39, no. 3 (March 2019): 439–464, 10.1017/s0144686x17000885.

[29] S. C. Olesen , P. Butterworth , and B. Rodgers , “Is Poor Mental Health a Risk Factor for Retirement? Findings From a Longitudinal Population Study,” Social Psychiatry and Psychiatric Epidemiology 47, no. 5 (2012): 735–744, 10.1007/s00127-011-0375-7. 21461932

[30] G. Di Gessa , L. Corna , D. Price , and K. Glaser , “Lifetime Employment Histories and Their Relationship With 10‐Year Health Trajectories in Later Life: Evidence From England,” European Journal of Public Health 30, no. 4 (August 2020): 793–799, 10.1093/eurpub/ckaa008. 32091579 PMC7445040

[31] P. Butterworth , S. C. Gill , B. Rodgers , K. J. Anstey , E. Villamil , and D. Melzer , “Retirement and Mental Health: Analysis of the Australian National Survey of Mental Health and Well‐Being. Social Science & Medicine,” Part A: Medical Sociology 62, no. 5 (2006): 1179–1191, 10.1016/j.socscimed.2005.07.013. 16171915

[32] P. Butterworth , C. Pymont , B. Rodgers , T. D. Windsor , and K. J. Anstey , “Factors That Explain the Poorer Mental Health of Caregivers: Results From a Community Survey of Older Australians,” Australian and New Zealand Journal of Psychiatry 44, no. 7 (2010): 616–624, 10.3109/00048671003620202. 20560849

[33] S. McManus , P. E. Bebbington , R. Jenkins , et al., “Data Resource Profile: Adult Psychiatric Morbidity Survey (APMS),” International Journal of Epidemiology 49, no. 2 (2020): 361–362e, 10.1093/ije/dyz224. 31725160

[34] N. Spiers , T. Qassem , P. Bebbington , et al., “Prevalence and Treatment of Common Mental Disorders in the English National Population, 1993–2007,” British Journal of Psychiatry 208, no. 2 (2016): 1–7, 10.1192/bjp.bp.115.174979. 27284080

[35] G. Lewis , A. J. Pelosi , R. Araya , and G. Dunn , “Measuring Psychiatric Disorder in the Community: A Standardized Assessment for Use by Lay Interviewers,” Psychological Medicine 22, no. 2 (1992 May): 465–486, 10.1017/s0033291700030415. 1615114

[36] K. Vo , P. M. Forder , M. Tavener , et al., “Retirement, Age, Gender and Mental Health: Findings From the 45 and Up Study,” Aging & Mental Health 19, no. 7 (July 2015): 647–657, 10.1080/13607863.2014.962002. 25271125

[37] C. E. Ross and P. Drentea , “Consequences of Retirement Activities for Distress and the Sense of Personal Control,” Journal of Health and Social Behavior 1, no. 4 (December 1998): 317–334, 10.2307/2676341. 9919854

[38] V. Richardson and K. M. Kilty , “Adjustment to Retirement: Continuity vs. Discontinuity,” International Journal of Aging and Human Development 33, no. 2 (September 1991): 151–169, 10.2190/6rpt-u8gn-vucv-p0tu. 1955209

[39] G. Mein , P. Martikainen , H. Hemingway , S. Stansfeld , and M. Marmot , “Is Retirement Good or Bad for Mental and Physical Health Functioning? Whitehall II Longitudinal Study of Civil Servants,” Journal of Epidemiology & Community Health 57, no. 1 (January 2003): 46–49, 10.1136/jech.57.1.46. 12490648 PMC1732267

[40] J. E. Kim and P. Moen , “Retirement Transitions, Gender, and Psychological Well‐Being: A Life‐Course, Ecological Model,” Journals of Gerontology: Series B 57, no. 3 (2002): P212–P222, 10.1093/geronb/57.3.P212. 11983732

[41] N. Steiber and M. Kohli , “You Can’t Always Get What You Want: Actual and Preferred Ages of Retirement in Europe,” Ageing and Society 34, no. 2 (2015): 1–385, 10.1017/s0144686x15001130.

[42] B. I. Mandal and B. Roe , “Job Loss, Retirement and the Mental Health of Older Americans,” Journal of Mental Health Policy and Economics 11, no. 4 (December 2008): 167–176. 19096091

[43] S. Adam , C. Bay , E. Bonsang , S. Germain , S. Perelman , “Occupational Activities and Cognitive Reserve: A Frontier Approach Applied to the Survey on Health, Ageing, and Retirement in Europe (SHARE)” (CREPP Working Paper 2006/05, Centre de Recherche en Economie Publique et de la Population (CREPP) (Research Center on Public and Population Economics) HEC‐Management School, University of Liège, 2006).

[44] S. Rohwedder and R. J. Willis , “Mental Retirement,” Journal of Economic Perspectives 24, no. 1 (2010): 119–138, 10.1257/jep.24.1.119. 20975927 PMC2958696

[45] N. Coe , H. M. von Gaudecker , M. Lindeboom , J. Maurer , “The Effect of Retirement on Cognitive Functioning” (Netspar Discussion Paper 10/2009‐044, 2009). 10.1002/hec.1771 21818822

[46] E. Whitley and F. Popham , “Leaving the Labour Market Later in Life: How Does It Impact on Mechanisms for Health?,” Occupational and Environmental Medicine 74, no. 12 (2017): 877–886, 10.1136/oemed-2016-104258. 28827279 PMC5740548

[47] E. Courtin , M. Knapp , and S. isolation , “Loneliness and Health in Old Age: A Scoping Review,” Health and Social Care in the Community 25, no. 3 (2015): 799–812. 26712585 10.1111/hsc.12311

[48] A. D. Ong , B. N. Uchino , and E. Wethington , “Loneliness and Health in Older Adults: A Mini Review and Synthesis,” Gerontology 62, no. 4 (2016): 443–449, 10.1159/000441651. 26539997 PMC6162046

[49] A. Schinkel‐Ivy , I. Mosca , and A. Mansfield , “Factors Contributing to Unexpected Retirement and Unemployment in Adults Over 50 Years Old in Ireland,” Gerontology and Geriatric Medicine 3 (2017): 2333721417722709, 10.1177/2333721417722709. 28808669 PMC5536377

[50] V. Patel , R. Araya , N. Chowdhary , et al., “Detecting Common Mental Disorders in Primary Care in India: A Comparison of Five Screening Questionnaires,” Psychological Medicine 38, no. 2 (February 2008): 221–228, 10.1017/S0033291707002334. 18047768 PMC4959557

[51] T. Abdeta , A. Birhanu , H. Kibret , et al., “Prevalence of Common Mental Disorders and Associated Factors Among Adults Living in Harari Regional State, Eastern Ethiopia: A Community Based Cross‐Sectional Study,” Frontiers in Psychiatry 14 (2023): 1183797, 10.3389/fpsyt.2023.1183797. 37520233 PMC10372418

[52] R. P. Cornish , A. John , A. Boyd , K. Tilling , and J. Macleod , “Defining Adolescent Common Mental Disorders Using Electronic Primary Care Data: A Comparison With Outcomes Measured Using the CIS‐R,” BMJ Open 6, no. 12 (2016): e013167, 10.1136/bmjopen-2016-013167. PMC5168670 27909036

[53] J. Head , S. A. Stansfeld , K. P. Ebmeier , et al., “Use of Self‐Administered Instruments to Assess Psychiatric Disorders in Older People: Validity of the General Health Questionnaire, the Center for Epidemiologic Studies Depression Scale and the Self‐Completion Version of the Revised Clinical Interview Schedule,” Psychological Medicine 43, no. 12 (December 2013): 2649–2656, 10.1017/S0033291713000342. 23507136 PMC3821376

[54] G. Di Gessa , L. Corna , D. Price , and K. Glaser , “The Decision to Work After State Pension Age and How It Affects Quality of Life: Evidence From a 6‐Year English Panel Study,” Age and Ageing 47, no. 3 (May 2018): 450–457, 10.1093/ageing/afx181. 29329400 PMC5920338

[55] H. Van Solinge and K. Henkens , “Couples' Adjustment to Retirement: A Multi‐Actor Panel Study,” Journals of Gerontology Series B: Psychological Sciences and Social Sciences 60, no. 1 (2005): S11–S20, 10.1093/geronb/60.1.S11. 15643041

